# Revealing the Diversity of Introduced* Coffea canephora* Germplasm in Ecuador: Towards a National Strategy to Improve Robusta

**DOI:** 10.1155/2017/1248954

**Published:** 2017-10-30

**Authors:** Rey Gastón Loor Solórzano, Fabien De Bellis, Thierry Leroy, Luis Plaza, Hilton Guerrero, Cristian Subia, Darío Calderón, Fabián Fernández, Iván Garzón, Diana Lopez, Danilo Vera

**Affiliations:** ^1^Instituto Nacional de Investigaciones Agropecuarias (INIAP), Programa Nacional de Cacao y Café (PNCC), Estación Experimental Tropical Pichilingue (EETP), Km 5 Vía Quevedo-El Empalme, Mocache, Los Ríos, Ecuador; ^2^CIRAD, UMR AGAP, 34398 Montpellier, France; ^3^AGAP, Université de Montpellier, CIRAD, INRA, Montpellier SupAgro, Montpellier, France; ^4^Instituto Nacional de Investigaciones Agropecuarias (INIAP), Programa Nacional de Cacao y Café (PNCC), Estación Experimental Central Amazónica (EECA), San Carlos, Joya de Los Sachas, Orellana, Ecuador; ^5^Instituto Nacional de Investigaciones Agropecuarias (INIAP), Departamento Nacional de Biotecnología (DNB), Estación Experimental Tropical Pichilingue (EETP), Km 5 Vía Quevedo-El Empalme, Mocache, Los Ríos, Ecuador; ^6^Instituto Nacional de Investigaciones Agropecuarias (INIAP), Departamento de Producción, Venta de Bienes y Servicios Agropecuarios (DPVBSA), Estación Experimental Tropical Pichilingue (EETP), Km 5 Vía Quevedo-El Empalme, Mocache, Los Ríos, Ecuador; ^7^Instituto Nacional de Investigaciones Agropecuarias (INIAP), Departamento Nacional de Protección Vegetal (DNPV), Estación Experimental Tropical Pichilingue (EETP), Km 5 Vía Quevedo-El Empalme, Mocache, Los Ríos, Ecuador; ^8^Universidad Técnica Estatal de Quevedo (UTEQ), Facultad de Ciencias Pecuarias, La María, Km 8 Vía Quevedo-El Empalme, Mocache, Los Ríos, Ecuador

## Abstract

Genetic resources of* Coffea canephora* have been introduced in several tropical countries with potential for crop development. In Ecuador, the species has been cultivated since the mid-20th century. However, little is known about the diversity and genetic structure of introduced germplasm. This paper provides an overview of the genetic and phenotypic diversity of* C. canephora* in Ecuador and some proposals for implementing a breeding program. Twelve SSR markers were used to analyze 1491 plants of* C. canephora* grown in different living collections in Ecuador, compared to 29 genotypes representing the main genetic and geographic diversity groups identified within the species. Results indicated that most of the genotypes introduced are of Congolese origin, with accessions from both main subgroups, SG1 and SG2. Some genotypes were classed as hybrids between both subgroups. Substantial phenotypic diversity was also found, and correlations were observed with genetic diversity. Ecuadorian Robusta coffee displays wide genetic diversity and we propose some ways of improving Robusta in Ecuador. A breeding program could be based on three operations: the choice of elite clones, the introduction of new material from other countries (Ivory Coast, Uganda), and the creation of new hybrid material using genotypes from the different diversity groups.

## 1. Introduction

*Coffea canephora* is originated from the lowland tropical forests of Africa, which stretch from Guinea to Uganda, and its cultivation is recent (end of the 19th century). Robusta coffee fields are now widely found in all lowland intertropical regions of Africa, America, and Asia [1]. The genetic diversity of* C. canephora* was first described at molecular level in the 1980s [1–7]. Those studies revealed two main diversity groups, the Congolese and the Guinean groups (G). The Congolese group was subdivided into five subgroups (SG1, SG2, B, C, and UW). Only a small portion of this wide diversity (i.e., mainly SG1 and SG2) is used in current breeding programs, with the exception of the recurrent breeding program in Ivory Coast, which uses a larger share of this diversity, except that from Uganda [4, 8, 9]. A core collection encompassing a large share of known* C. canephora* diversity has been recently proposed [10]. This core collection contains genotypes from all the known diversity groups and is an interesting starting point from which to broaden genetic diversity in* C. canephora* breeding programs.

In Ecuador,* C. canephora* genetic resources were first introduced in the mid-20th century [11, 12]; the origin of this germplasm is diverse but little information is available on its true geographic origin or its diversity and genetic structure. This information is considered very important for future conservation and development conditions for a breeding program in the country.

Ecuadorian historical records show that the first introductions of* C. canephora* genetic material came from the “Tropical Agricultural Research and Higher Education Center (CATIE),” Costa Rica, in 1951, 1964, 1972, 1977, and 1986. They corresponded to the “*Robusta*” type (putative SG2) and all were planted at the Pichilingue Tropical Research Station (EETP) of the National Institute of Agricultural Research of Ecuador (INIAP), with the first* C. canephora* plantations appearing in 1952 in Los Ríos province, from where they were gradually extended to several coastal provinces and toward the north of Ecuadorian Amazonia [13].

Later, in 1987 and 2006, genetic material of the “Conilon” type (putative SG1) was imported from Brazil. Additionally, unofficial sources report introductions of genetic material imported as seeds from Vietnam and Indonesia (2009, 2010), as well as Robusta from Brazil (2010). Using seeds for genetic material transfers for a self-incompatible tree cannot ensure its genetic origin since crosses between genotypes from different genetic groups are likely to occur within germplasm collections [10].

During the second half of the 20th century, a nonspecific* C. canephora* breeding program was developed. However, a first group of elite material “clones” was identified by INIAP in 1998, based on yield and morphological traits. To date, these clonal* C. canephora* materials have been recommended for commercial planting under the conditions of northern Ecuadorian Amazonia. A recent study on the phenotypic characterization of* C. canephora* accessions planted in the living genebank collection, located in the EETP of the INIAP, showed a high variability between and within these accessions [13]. Consequently, knowledge on the genetic diversity of the material widely distributed in the Ecuadorian territory could help breeders and geneticists to understand the structure of introduced germplasm in order to design a* C. canephora* breeding program in Ecuador.

This paper will

- provide an overview of the genetic and phenotypic diversity and conservation of* C. canephora* in Ecuador,
- address the bases for implementing a* C. canephora* breeding program.

## 2. Material and Methods

### 2.1. Plant Material

In all, 1491 leaf samples were taken from different* C. canephora* clonal plants collected during 2011, 2012, and 2013. In 2011, one hundred thirty-seven leaf samples were collected from two fields. The first field is the Dublinsa-Denisse Farm, nearby Isidro Ayora in Guayas province that has a living collection of* C. canephora* collected from different locations at the Amazonian region of Ecuador. The second field is located at the EETP research station, nearby Quevedo city, Los Rios province. In 2012, two hundred leaf samples from 12 clonal accessions and 154 leaf samples from seven clonal accessions and a polyclonal mix were collected at the Dublinsa-Denisse Farm and the EETP, respectively. In 2013, one thousand leaf samples from 7 clones were sampled at the INIAP's Amazonian Central Station (EECA).

The summary of the plant material used for genotypic analysis across the different years is shown in Tables 1(a) and 1(b). Among these, 48 samples were considered as duplicates and used to check the experimental reproducibility of the data and accordingly homogenize the data whenever needed.

### 2.2. DNA Preparation and Genotyping

Genomic DNA extractions were performed according to Cubry et al. [14]. The 1491 accessions were genotyped with the 12 SSR markers used by Leroy et al. [10]. Two different methods were used for genotyping and allele calling in 2011 and 2012/2013, respectively.

In 2011, PCR reactions ran as described in Cubry et al. [14]. PCR products were analyzed by electrophoresis on a 6.5% polyacrylamide gel using a LI-COR 4300 automated sequencer (LI-COR Biosciences, Nebraska, USA). Size calling was automatic and manually checked using the manufacturer's program SAGA^GT^.

In 2012 and 2013, PCR reactions were performed according to De Bellis et al. [15]. In a solution A (25 *μ*L total volume) containing 2.5 *μ*L of PCR buffer (10 mM Tris-HCl, 50 mM KCl, 2 mM MgCl2, and 0.001% glycerol), 2.5 *μ*L of dNTP (Jena Bioscience GmbH, Jena, Germany), 0.25 *μ*L of MgCl2, 0.2 *μ*L of 10 *μ*M forward primer with an M13 tail at the 5′-end (5′-CACGACGTTGTAAAACGAC-3′), 0.25 *μ*L of 10 *μ*M reverse primer, 0.25 *μ*L of fluorescently labeled M13-tail (6-FAM, NED, VIC, or PET from Applied Biosystems, Foster City, California, USA), 0.1 U of Taq DNA polymerase (Sigma, St. Louis, Missouri, USA), 5 *μ*L of template DNA (5 ng/*μ*L), and 14 *μ*L of H20. The PCR conditions were as follows: an initial denaturation at 94°C for 5 min; 30 cycles at 94°C for 45 s, 55°C for 45 s, and 72°C for 1 min; and a final extension at 72°C for 10 min. PCR products were pooled in a solution B containing 2 *μ*L of 6-FAM, 2 *μ*L of VIC, 2.5 *μ*L of NED, and 3.5 *μ*L of PET of A's solutions. From this solution B, a volume of 4 *μ*L was taken and added to 10 *μ*L of Hi-Di formamide and 0.12 *μ*L of GeneScan 600 LIZ size standard and analyzed on an ABI 3500xL Genetic Analyzer (Life Technologies, Carlsbad, California, USA). Alleles were scored using GeneMapper v4.1 software (Applied Biosystems, Foster City, California, USA).

### 2.3. Data Analysis

#### 2.3.1. Whole of Ecuador Genetic Analyses

Data were obtained separately in 2011, 2012 and 2013 for each sampling year, from which merged three matrices. Standard controls of known genotypes and duplicates samples together with genotyping database (SagaCityWeb, CIRAD, unpublished) were used to help us to pool/shift alleles to ensure good coherence of the genotyping data. From the raw data, were removed the 48 duplicates and the samples with more than 17% of missing data (i.e., two missing markers) for further analysis. The number of alleles detected per marker was recorded and compared to those detected in Leroy et al. [10]. We computed dissimilarity matrices between individuals using a simple matching index with Darwin 6.0 software [16].

A first diversity tree was drawn up using data from the 2011 sampling operation in Ecuador. Diversity from Ecuador was evaluated in relation to the global diversity of the species.

A global analysis, using data from the three sampling years, was performed to identify unique genotypes for future conservation and for breeding programs. In all, 1168 samples were kept for analysis, based on missing values (<8% not available, i.e., one marker missing). A Neighbor-Joining (NJ) tree was built on the whole data removing pairs of data with more than 70% of missing values. To take into account the noise due to highly repeated genotypes, the max-length subtree procedure was used to eliminate redundancy and to identify the number of unique individuals without loss in the number of alleles. A Principal Coordinates Analysis (PCoA) was used to construct a good image of the diversity between genotypes.

Specific analyses were performed at the DUBLINSA and EECA stations to check the homogeneity and the diversity of the materials introduced.

#### 2.3.2. The EETP Collection

Phenotypic analysis was performed at the EETP collection, using data from 2010 to 2012. The evaluation was carried out in 256 plants, corresponding to 16 accessions. Each accession contained different number of individual (between 12 and 20). A phenotypic dendrogram was performed by UPGMA clustering method using the Euclidean distance.

Genotyping data were available for 146 plants. Tree diversity was determined for molecular data, and their relationship with phenotypic data generated by Plaza et al. [13] was studied.

Phenotypic data were measured between 2010 and 2012 for all the trees planted in 2007. During this time, the traits observed were the following: plant height (PH), stem diameter (SD), number of branches (NB), number of nodes per branch (NN), and internode length (IL). Outlier data were removed whenever found and replaced the missing values. PCA analyses were performed using the* dudi.pca* function from the ade4 R package [17]. Five principal components were chosen after observing the screen plot of eigenvalues. Correlation circle was drawn using the* s.corcircle* function and the representation of individuals for the first two components was drawn using the* s.class* function.

Two types of analyses were performed on these data. A clustering tree was first built on 249 plants using the dissimilar Euclidian distance evaluated by average linkage for the 17 traits observed across the three years. Another analysis was performed using a Principal Components Analysis (PCA) on five vigor traits (yield of cherry beans, number of cushion flowers, total of productive branches, total of branches per tree, and tree height) across three years observed on the 146 trees analyzed for genetic diversity.

## 3. Results

### 3.1. Whole of Ecuador Genetic Analyses

**Figure 1:**
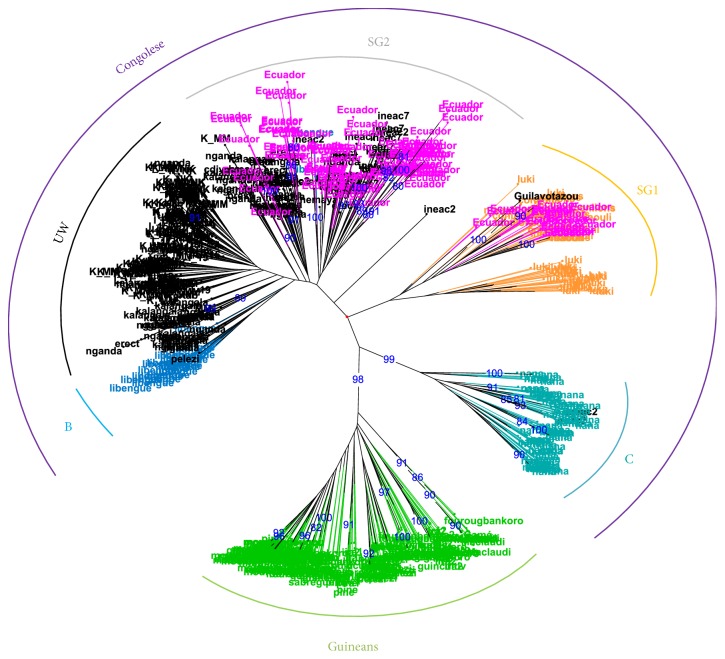
Genetic diversity of 137 Ecuadorian* Coffea canephora* accessions within the known diversity of the species (NJ-Tree from data produced in 2011).

The results on the clonal trials at the EETP and Dublinsa revealed great diversity between “clones,” and also a high level of diversity within “clones,” which was more surprising. The global genetic analysis indicated the Congolese origin for all the genotypes. They were classed as SG1 or SG2 genotypes, with some hybrids between these two Congolese subgroups. This result can be explained by considering the origin of plants introduced in Ecuador: the SG1 genotypes could have come from Brazil (Conilon genotypes are known to be from the SG1 group) and the SG2 genotypes from CATIE (from the Congo basin).  Figure 1 presents the diversity tree identifying the genotypes from Ecuador within the global diversity of* C. canephora*.

**Figure 2:**
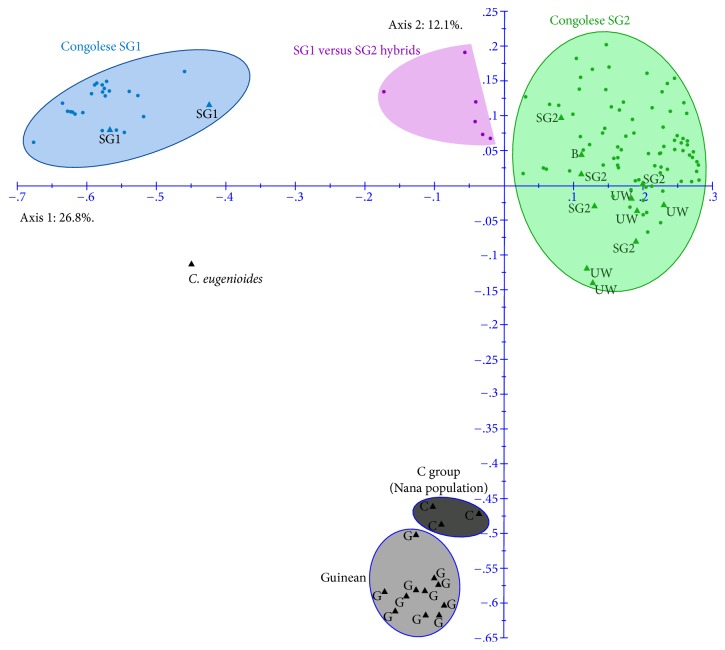
Principal coordinates analysis based on the dissimilarity matrix, calculated using 12 SSR markers for 138 individual genotypes (29 controls and 109 genotypes from Ecuador).

A global analysis was performed in 2015, based on the results obtained for the 1168 accessions, by considering all the different genotypic classes revealed for each year in each plot. A global diversity tree was established using these 1168 accessions (figure not shown). After eliminating redundancy, the 138 unique genotypes (including 29 controls) were observed in the diversity tree (figure not shown). The PCoA analysis performed 138 unique genotypes, most of these genotypes were included in the SG1 and SG2 diversity groups, and six genotypes could be considered as hybrids between the groups (Figure 2).

**Figure 3:**
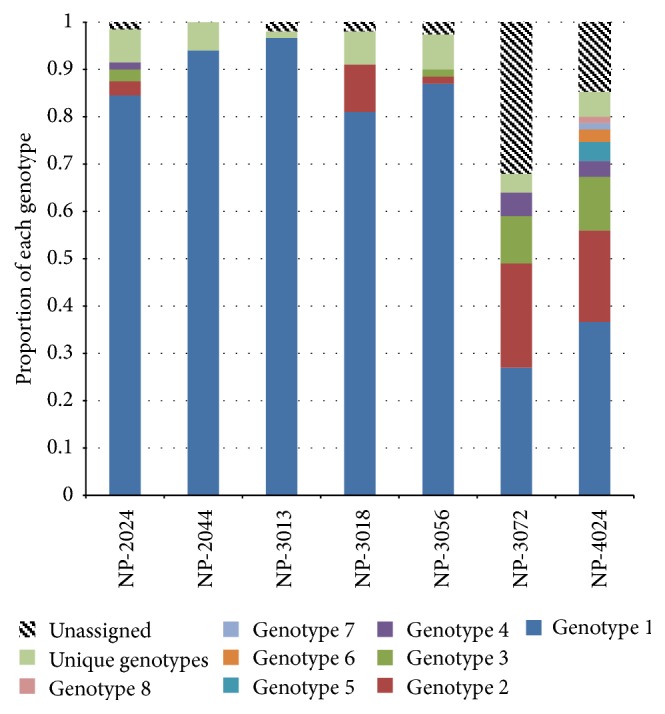
Diagram bars showing the mixture within the 7 “clonal accessions” from INIA's EECA experimental station. For comparison purposes, data are expressed as a percentage of plants per “clone.”

**Figure 4:**
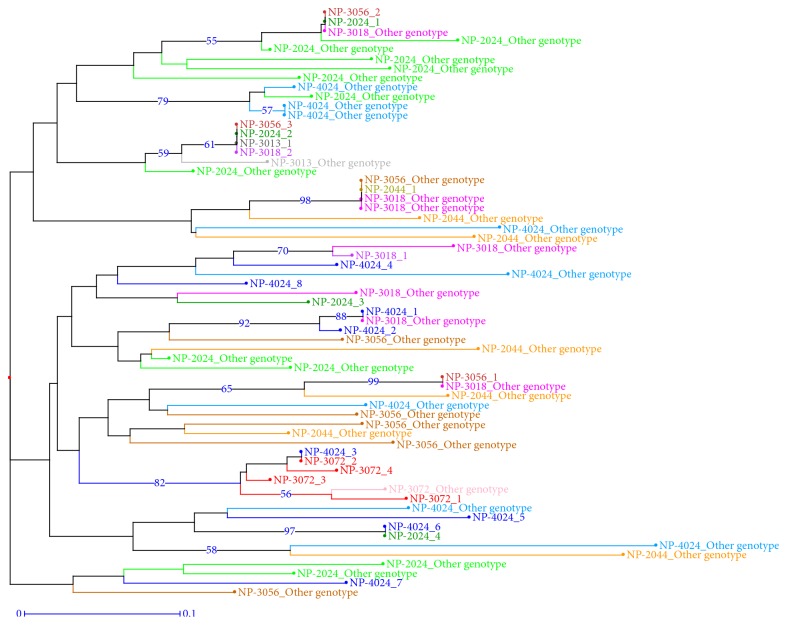
Dissimilarity analysis of 7 “clonal accessions” from the EECA station in 2013. NJ-Tree on the 63 different unique genotypes using 12 SSR markers. Branch support is expressed in percent of presence after 10000 bootstraps (values above 0.5 are displayed).

Figure 3 shows the results for the genotypic analysis, considering the “clones” analyzed in 2013 at the EECA station. For each accession/clone, 2 to 8 genotypic classes were identified. Accessions NP-3013 and NP-2044 only had one genotype. Some genotype classes were similar for certain accessions: genotype 1 from NP-2024 was similar to genotype 2 for NP-3056; genotype 1 from NP-3013 was similar to genotype 2 for NP-3018 and NP-2024 and also to genotype 3 for NP-3056; genotype 2 from NP-3072 was similar to genotype 3 for NP- 4024; genotype 4 from NP-2024 was similar to genotype 6 for NP-4024. The dissimilarity tree at the EECA living collection (Figure 4) yielded 50 genotyping classes. Several classes grouped individuals belonged to different accessions.

### 3.2. EETP Collection

**Figure 5:**
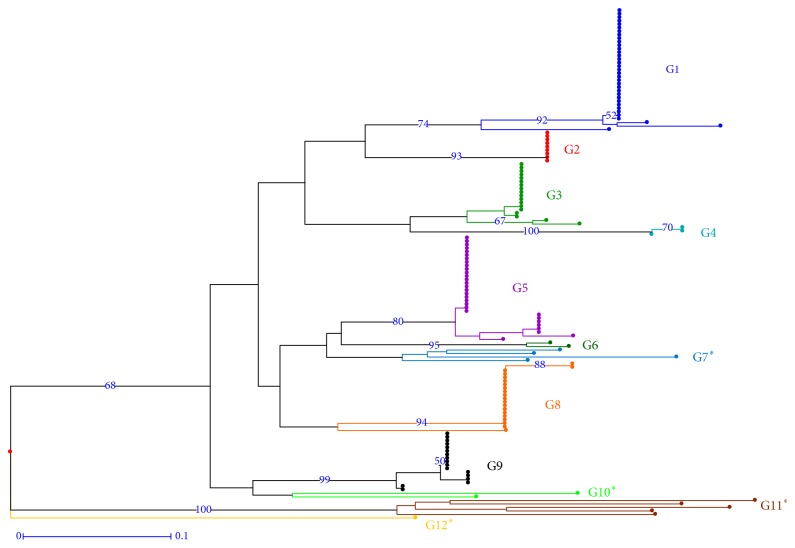
Diversity tree for the 146 plants analyzed at the Pichilingue collection in 2012.

A diversity tree was constructed considering the 146 plants from the 16 different origins with data available for molecular diversity. Twelve diverse groups were identified within the 146 (from 154) plants analyzed in 2012 (Figure 5).

On the other hand, a phenotypic characterization of this collection was reported by Plaza et al. [13] showing a wide range of variation in most of the agro-morphological traits evaluated for each plant. The existence at this level of phenotypic variation was found between and also within accessions. This phenotypic result was the first to open up the possibility of off-types in this collection; despite this, 25 plants were selected as “elite” material, but the most important variations considered when selecting those elite plants were plant yield and plant height [13]. These 25 plants (24 analyzed) were identified by their genetic diversity based on two analyses, per origin and individually:

- Per origin analysis: COF1 p2 was different from COF1 p10; COF3 p2, 5, 8, 18, and 19 were genetically similar; p7 was different; COF4 p7 and 9 were similar; p15 was different. COF 5 p6, 15, 16, 17, and 19 were similar; NP2024 p 7, 10, 15, and 17 were similar; NP2044 p6, 16, and 17 were similar; NP3018 p 8 and 19 were probably different, since the origin was very variable.
- Global genetic diversity of the 24 “elite” clones: COF1 p10, COF3 p2, 5, 8, 18, and 19, and COF4 p7 and 9 were similar; COF5 p6, 15, 16, 17, and 19 and NP2024 p7, 10, 15, and 17 were similar; NP2044 p6, 16, and 17 were similar; COF4 p15 and COF3 p7 were similar; COF1 p2 and NP3018 p19 were almost identical; their similarity has to be verified.

The genetic diversity of the selected clones was therefore very low, since only 5 different groups of genotypes (six if including NP3018) were present in the selected clones. As regards the global diversity of the Pichilingue collection, almost half of this diversity was not present in the selected clones. The diversity of the Conilon/SG1 group was not present in the selected clones from this collection.

### 3.3. Phenotypic Diversity

**Figure 6:**
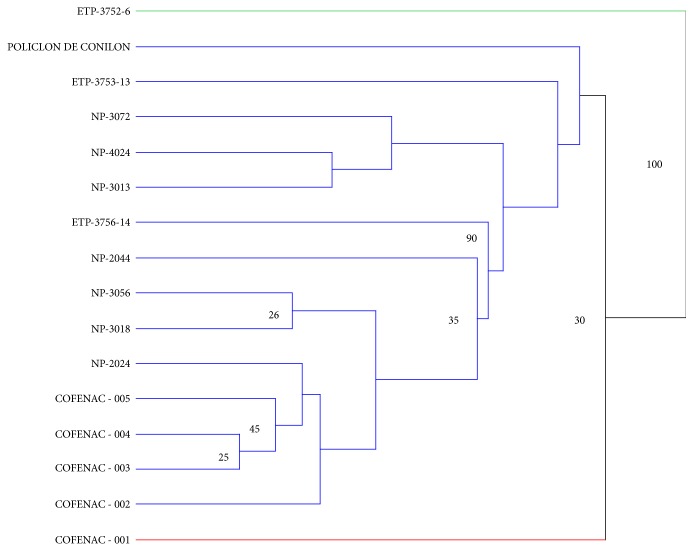
Phenotypic dendrogram for 16 accessions at EETP* C. canephora* collection. The data were collected between 2010 and 2012 using vigor traits (productivity, stem diameter, plant height, number of branches, number of nodes, and internode length). Branch support is expressed in percent of presence after 10000 bootstraps (values above 0.2 are displayed).

The phenotypic dendrogram at the EETP* C. canephora* collection (Figure 6) indicated 3 clusters. One cluster (group I) included 14 accessions with a high diversity within the group. The second and third group consisted of one isolated accession, respectively.

**Figure 7:**
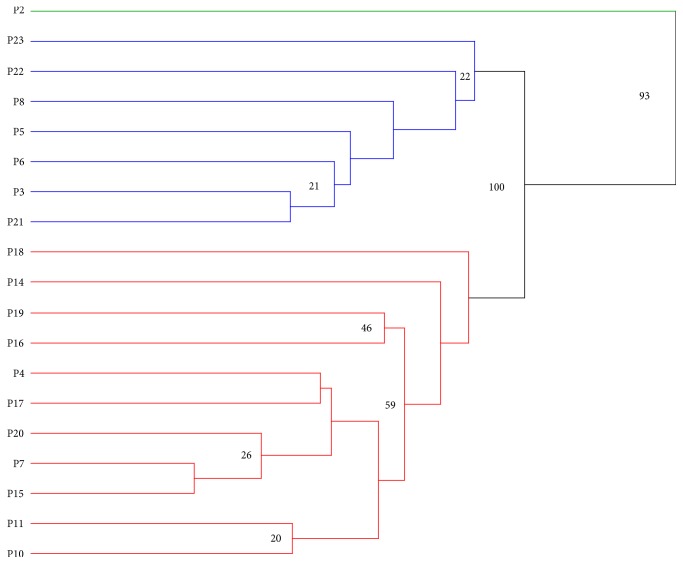
Phenotypic dendrogram for 19 individuals belonging to NP-2024 accession at EETP* C. canephora* collection. The phenotypic data were collected between 2010 and 2012 for vigor traits (productivity, stem diameter, plant height, number of branches, number of nodes, and internode length). Branch support is expressed in percent of presence after 10000 bootstraps (values above 0.2 are displayed).

Phenotypic results were highly variable in most plants belonging to the same accession. To determine the level of this variation, all individuals from NP2024 accession were used as sample. The dendrogram indicated 3 clusters, confirming the high level of variability among the individuals of this accession (Figure 7).

**Figure 8:**
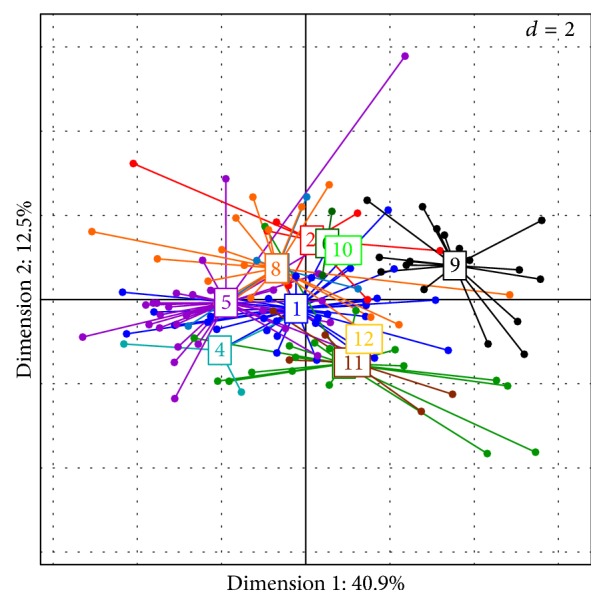
PCA for the 146 genotypes in the EETP collection for the vigor traits in 2012.

A complemented phenotypic PCA using the vigor traits was carried out with the 12 genetic groups previously identified at the EETP collection (i.e., Figure 5) showing a significant phenotypic diversity within of the groups (Figure 8). Group 7 was considered as presenting short internodes, and group 9 exhibited low vigor and short internodes, while group 5 had rather long internodes and high vigor, and group 11 was characterized by low vigor and long internodes.

**Figure 9:**
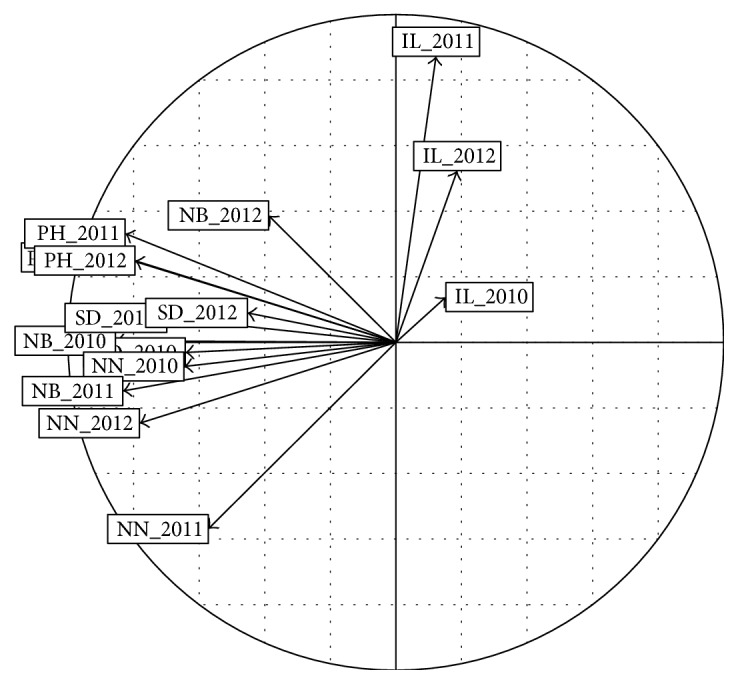
PCA correlation circle for phenotypic vigor traits over 3 years, 2010, 2011, and 2012, for 146* C. canephora* trees in the field collection at the EETP.

On the other hand, using PCA, the correlation circle between observed traits (Figure 9) indicated that the internode length (IL) for the three years was related to axis 2, since the other vigor traits, particularly stem diameter (SD), plant height (PH), and the total number of nodes per tree (NNT) were related to axis 1.

## 4. Discussion

Our analyses

- confirmed the genetic diversity of accessions from Ecuador, covering the SG1 and SG2 subgroups of Congolese diversity, in accordance with the history of introductions,
- revealed great genetic and phenotypic diversity between clones, but also a large number of genotype classes within most of the “clonal” accessions,
- suggested some ways of implementing a breeding strategy for* C. canephora* using the available diversity.

The first point concerns the reliability of our work for the three-year experiment. The first-year analyses were performed using LI-COR 4300 technology. The following analyses were performed using an ABI sequencer, with different control plants. Misidentification of alleles during calling and binning processes are known caveats of SSR studies [18]. To solve this issue, laboratory good practices were implemented by including controls of known genotypes and by repeating some samples from one study to another. The overall results are in accordance with what was expected and thus could be considered of good quality.

It was a challenge to analyze concomitantly all the data for the final evaluation. The results could thus be considered as a compromise between all the different data. However, the low quality of some leaf material was also an element that we took into account, due to difficulties for DNA extraction and analysis with microsatellites. Some leaves samples were not correctly analyzed and were removed from the final analyses.

In 2012, at DUBLINSA collection, we detected genetic diversity within “clones” in our analyses, meaning that the “clones” were not genetically homogeneous, as they ought to have been (data not shown). We also observed mixtures between genotypes in both clonal trials. The CONERBO and POLICLON genotypes which belonged to Conilon type can be considered as plants from a mix of seeds, introduced from Brazil in the 80s; these origins present high genetic diversity. Regarding the EETP collection, it should be noted that many plants, with different labels, were quite similar from a genetic point of view. We also had to consider that the Conilon exhibited wide diversity, possibly due to its environmental share of diversity or due to their seed origin.

In 2014, a global analysis of* C. canephora* diversity was carried out, using a core collection approach [10]. The comparison with the present study confirmed that the diversity observed within Ecuadorian accessions accounts for about 57% of core collection diversity, considering the different alleles. Therefore, the information provided by this study will help breeders choose the most appropriate plant(s) or accession(s) to be incorporated into their breeding programs.

Another finding was the small number of intergroup hybrids between the SG1 and SG2 diversity groups. This low level of hybridization can be explained by the history of the introduced material. Both introductions were composed of genotypes from a single subgroup (SG2 from CATIE and SG1 from Brazil). The accessions were planted in one location (EETP) and then transferred to Manabí, Santo Domingo de los Tsáchilas, and Morona Santiago provinces, mainly by cuttings. Thus, few hybrids can be found between groups in the Ecuadorian collections.

Nevertheless, high diversity was observed within each diversity group (SG1 and SG2). For SG2, this diversity was related to the large number of accessions that were introduced, and previous studies [7] confirmed the high diversity within this group. The SG1 genotypes were mainly introduced by seeds, for which diversity is always greater than the clones in this allogamous species. High genetic and phenotypic diversity within this group has been recently established. In the case of the Pichilingue germplasm, it should be noted that high levels of phenotypic variation were previously reported by Plaza et al. [13] and could be related to the continuous pollen interchange and/or possible mix of seeds from different segregated populations.

The phenotypic characterization carried out by Plaza et al. [13] and our study enabled us to identify a wide range of variation in most of the agro-morphological traits evaluated per plant, with significant phenotypic diversity within genotypes. In this respect, it is important to keep in mind that a phenotype is a product of genotype × environment interaction, which we found in our results. Therefore, plants may be morphologically similar, but this does not necessarily imply genetic similarity, since different genetic bases can result in similar phenotypic expression [18] and, as observed in our results, the same genotype can lead to substantial differences in phenotypic expression.

In our view, a combined analysis of phenotypic and molecular marker results is crucial for a better understanding of evolutionary changes in this introduced species; this would allow a better analysis of variation patterns within* C. canephora* for evaluating their future adaptive potential in different geographical regions of Ecuador. Differences between phenotypic and genetic information have also been found in other crops [19–22].

Lastly, this study was intended to identify diversity and ways of using it to increase the production of Robusta coffee in Ecuador. In Ivory Coast, considering the high genetic diversity found in* C. canephora*, a program of reciprocal recurrent selection was conducted using the hybrid vigor observed between genotypes of different origins [8, 23, 24]. Based on the Ivorian experience, we might propose some steps for breeding* C. canephora* in Ecuador, with optimum use of the existing diversity and improved management of the existing material, based on the results presented. This strategy for the implementation of a* C. canephora* breeding program should also be of interest for other countries where coffee genotypes have been introduced in recent decades.

As a first step, we propose the following actions, using the diversity existing in Ecuador:

- To complete genotypic analyses with phenotypic data to increase knowledge on the accessions in the field for their vigor, productivity, and stress and disease tolerance
- To reorganize the collections based on the genetic diversity observed in our studies to avoid duplications and identifying unique genotypes
- To implement a breeding program by selecting the best genotypes for traits of interest (yield, biotic and abiotic stress tolerance, and adaptation to different edaphoclimatic conditions) (these genotypes will be planted as clones in a multisite trial design. Attention will be paid to the high diversity of plants from the Conilon/SG1 group. These clonal trials will enable the selection of a set of improved genotypes for farmers)

As a second step, we propose the following actions, by increasing genetic diversity:

- To introduce new genetic material from diverse groups (i.e., Guinean, Ugandan, and hybrids between them) that are not present in Ecuador and test them under Ecuadorian conditions
- To establish a breeding program based on new hybrids obtained from crosses between genotypes from different diversity groups, adapted to the edaphoclimatic conditions in Ecuador (these new hybrids will use accessions from Ecuador and introduced accessions to create new hybrids. This program will use the diversity of both the SG1 and SG2 groups, as hybrids between these groups display hybrid vigor and good drought tolerance, as already observed in Ivory Coast. All the new hybrids will be tested under all the conditions in Ecuador, and this will lead to a new selection of elite clones or hybrids. This improved material will be distributed to farmers through cutting gardens (for clones) or seed gardens (for hybrids))

Over the long term, hybrid selection might be the optimum breeding method, as seeds are more suitable for distribution to growers, and the nurseries are easier for farmers to manage.

## 5. Conclusions

The present research concluded that Ecuadorian Robusta coffee displays a wide genetic diversity between clones and also a high level of diversity within clones. This research confirms that most of the* C. canephora* genotypes introduced in Ecuador are of Congolese origin, containing accessions from both subgroups, SG1 and SG2.

## Figures and Tables

**Table 1a:** (a) Analyzed plants

Year	Locations	Number of samples
2011	Dublinsa-Denisse Farm	81
	EETP	56
	*Total 2011*	137

2012	Dublinsa-Denisse Farm	200
	EETP	154
	*Total 2012*	354

2013	EECA	1000

**Table 1b:** (b) Controls

Diversity group	Diversity subgroup	Number of samples
Guinean	G	11

Congolese	B	1
	C	3
	SG1	3
	SG2	5
	*UW*	5

Outgroup	*Coffea eugenioides*	1

## References

[1] Montagnon C., Leroy T., Yapo A. (1992). Diversité génotypique et phénotypique de quelques groupes de caféiers (*Coffea canephora* Pierre) en collection. *Café Cacao Thé*.

[2] Berthaud J. (1986). *Les ressources génétiques pour l'amélioration des caféiers africains diploïdes. Evaluation de la richesse génétique des populations sylvestres et de ses mécanismes organisateurs”. Conséquences pour l'application*.

[3] Dussert D., Lashermes P., Anthony F., Hamon P., Seguin M., Perrier and JC Glaszmann X. (1999). Le caféier, *Coffea canephora*. in: P Hamon, M Seguin, X Perrier and JC Glaszmann. *Diversité génétique des plantes tropicales cultivées*.

[4] Montagnon C. (2000). *“Optimisation des gains génétiques dans le schéma de sélection récurrente réciproque de Coffea canephora Pierre [Ph.D. thesis]*.

[5] Musoli P., Cubry P., Aluka P. (2009). Genetic differentiation of wild and cultivated populations: Diversity of Coffea canephora Pierre in Uganda. *Genome*.

[6] Gomez C., Dussert S., Hamon P., Hamon S., Kochko A. D., Poncet V. (2009). Current genetic differentiation of coffea canephora pierre ex a. Froehn in the guineo-Congolian african zone: Cumulative impact of ancient climatic changes and recent human activities. *BMC Evolutionary Biology*.

[7] Cubry P., de Bellis F., Pot D., Musoli P., Leroy T. (2013). Global analysis of Coffea canephora Pierre ex Froehner (Rubiaceae) from the Guineo-Congolese region reveals impacts from climatic refuges and migration effects. *Genetic Resources and Crop Evolution*.

[8] Leroy T., Montagnon C., Charrier A., Eskes A. B. (1993). Reciprocal recurrent selection applied to Coffea canephora Pierre. I. Characterization and evaluation of breeding populations and value of intergroup hybrids. *Euphytica*.

[9] Montagnon C., Leroy T., Eskes A. B. (1998). Amélioration variétale de Coffea canephora I. Critères et méthodes de sélection. *Plantations, Recherche, Developpement*.

[10] Leroy T., De Bellis F., Legnate H. (2014). Developing core collections to optimize the management and the exploitation of diversity of the coffee Coffea canephora. *Genetica*.

[11] INIAP (Instituto Nacional Autónomo de Investigaciones Agropecuarias) “Informe Anual Técnico”. Estación Experimental Tropical Pichilingue. Programa del Café. Quevedo – Ecuador. (1977)

[12] COFENAC (Consejo Cafetalero Nacional). “Informe Anual de la División Técnica”. Portoviejo – Ecuador. (2004)

[13] Plaza L. F., Loor R. G., Guerrero H. E., Duicela L. A. (2015). Phenotypic characterization of *Coffea canephora* Pierre germplasm for yield improvement in Ecuador. *Espamciencia*.

[14] Cubry P., Musoli P., Legnaté H. (2008). Diversity in coffee assessed with SSR markers: Structure of the genus Coffea and perspectives for breeding. *Genome*.

[15] De Bellis F., Malapa R., Kagy V., Lebegin S., Billot C., Labouisse J.-P. (2016). New Development and Validation of 50 SSR Markers in Breadfruit (Artocarpus altilis, Moraceae) by Next-Generation Sequencing. *Applications in Plant Sciences*.

[16] Perrier X., Jacquemoud-Collet J. P. http://darwin.cirad.fr/darwin.

[17] Dray S., Dufour A. B. (2007). The ade4 package: implementing the duality diagram for ecologists. * Journal of Statistical Software *.

[18] Guichoux E., Lagache L., Wagner S. (2011). Current trends in microsatellite genotyping. *Molecular Ecology Resources*.

[19] Mazzucato A., Ficcadenti N., Caioni M. (2010). Genetic diversity and distinctiveness in tomato (Solanum lycopersicum L.) landraces: The Italian case study of 'A pera Abruzzese'. *Scientia Horticulturae*.

[20] Wang J., Kaur S., Cogan N. O. I. (2009). Assessment of genetic diversity in Australian canola (Brassica napus L.) cultivars using SSR markers. *Crop & Pasture Science*.

[21] Terzopoulos P. J., Bebeli P. J. (2008). DNA and morphological diversity of selected Greek tomato (Solanum lycopersicum L.) landraces. *Scientia Horticulturae*.

[22] Vinu V., Singh N., Vasudev S. (2013). Assessment of genetic diversity in Brassica juncea (Brassicaceae) genotypes using phenotypic differences and SSR markers.. *Revista de Biología Tropical*.

[23] Leroy T., Montagnon C., Charrier A., Eskes A. B. (1993). Reciprocal recurrent selection applied to Coffea canephora Pierre - II. Estimation of genetic parameters. *Euphytica*.

[24] Leroy T., Montagnon C., Cilas C., Yapo A., Charmetant P., Eskes A. B. (1997). Reciprocal recurrent selection applied to Coffea canephora Pierre. III. Genetic gains and results of first cycle intergroup crosses. *Euphytica*.

